# SpheroQuaNNt: U-Net–based volume estimation in tumor spheroid and organoid models for assessing preclinical treatment response

**DOI:** 10.1007/s10616-026-01074-9

**Published:** 2026-10-25

**Authors:** Nathalia Leal Dovaizem, Jean Carlos dos Santos da Luz, Alexis Germán Murillo Carrasco, Luciana Nogueira de Sousa Andrade

**Affiliations:** 1 Center for Translational Research in Oncology (LIM24), Instituto do Cancer do Estado de Sao Paulo (ICESP), Hospital das Clinicas da Faculdade de Medicina da Universidade de Sao Paulo (HCFMUSP), São Paulo 01246-000, Brazil, São Paulo, Brazil; 2 Comprehensive Center for Precision Oncology, Universidade de São Paulo, São Paulo, 01246- 000 Brazil

**Keywords:** 3D cell culture, Tumor spheroids, Organoids, Volume estimation, Image segmentation, Convolutional neural network

## Abstract

Three-dimensional (3D) cell culture models, including tumor spheroids and organoids, more accurately recapitulate the structural and physiological complexity of tissues than conventional monolayer cultures, making them valuable platforms for cancer research and drug screening. Volume is a biologically relevant parameter for monitoring growth dynamics and treatment response; however, its quantification often relies on manual image analysis, which is labor-intensive, subjective, and prone to inter-operator variability. Here, we introduce SpheroQuaNNt, a web-based application for automated spheroid segmentation and volume estimation using a U-Net Convolutional Neural Network (CNN). SpheroQuaNNt enables analysis of heterogeneous morphologies through image-based segmentation and provides a web-based interface for volume measurement. The model was trained and validated using 615 microscopy images representing diverse 3D culture systems, including monoculture and hybrid spheroids generated from human and canine tumor cell lines and human embryonic kidney cells, as well as tumor organoids derived from human melanoma lesions. After 30 training epochs, the model achieved 99.35% accuracy during training and 98.56% on the validation dataset, with an average Dice Score of 0.9478 for supervised validation. The method accurately captured volume variations across heterogeneous spheroids, although those presenting marked density heterogeneity between the core and periphery with a dark peripheral margin remained more challenging. Estimated volumes showed strong agreement with measurements performed independently by two experts, supporting its reliability. Overall, SpheroQuaNNt provides a standardized solution for volumetric characterization of spheroids and organoids, reducing operator-dependent variability and supporting high-throughput drug screening and quantitative analysis of 3D cell culture models.

**Graphical abstract:**

**Figure:**
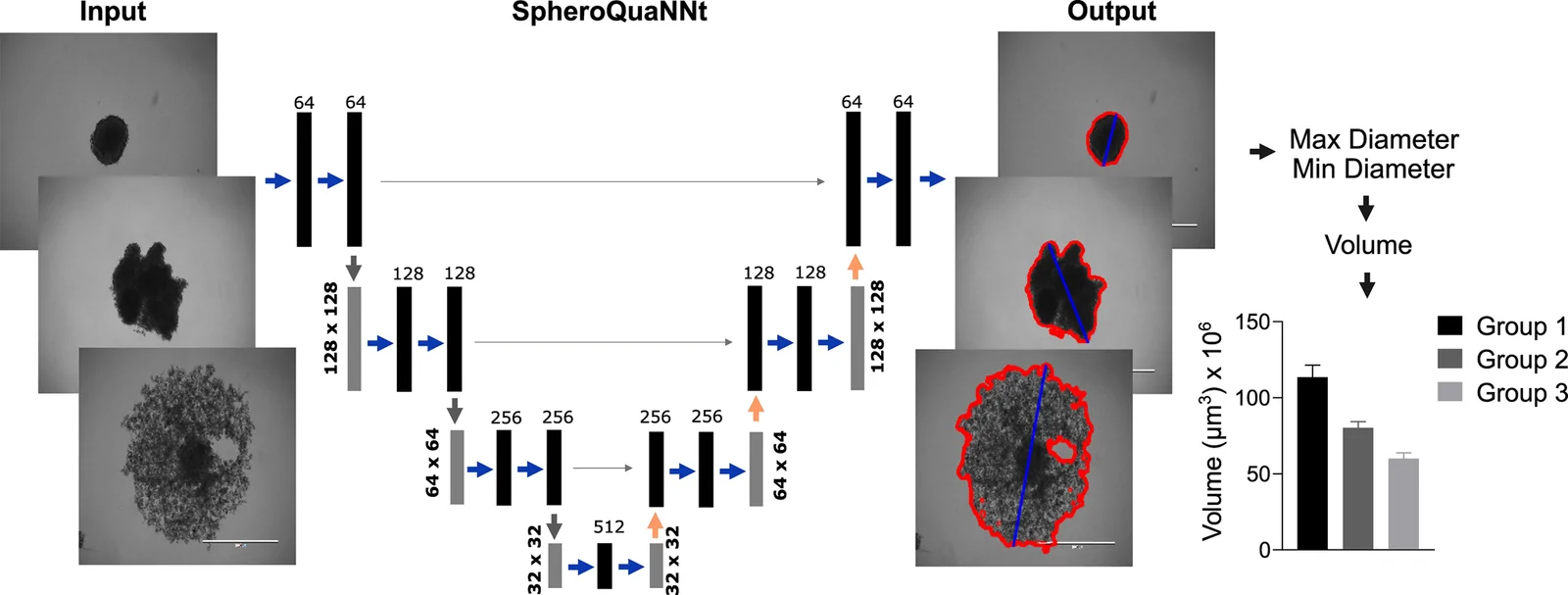

**Supplementary Information:**

The online version contains supplementary material available at https://doi.org/10.1007/s10616-026-01074-9.

## Introduction

Spheroids are widely used as three-dimensional tumor models due to their ability to more accurately recapitulate the tumor microenvironment when compared to bi-dimensional monolayer cultures, mimicking essential features such as cell–cell interactions, gradients of nutrients and oxygen, and differential drug penetration; which provides a physiologically relevant platform for the evaluation of novel therapies and combinatorial treatments (Li et al. 2023; Nayak et al. 2023; Shah and D’Souza 2025). Organoids, including patient-derived organotypic tumor spheroids (PDOTS), represent an advanced class of 3D culture system that preserve key genetic, phenotypic, and functional characteristics of the original tumors, further enhancing the translational relevance of in vitro models (Clevers 2016; Vlachogiannis et al. 2018; Drost and Clevers 2018; Bozym et al. 2025).

Although the therapeutic responses in these systems are commonly assessed by analyzing morphometric features from microscopy images, manual quantification remains a labor-intensive and operator-dependent task that limits throughput and introduces variability, ultimately compromising data reproducibility and accuracy. By enabling data-driven pattern recognition and prediction, machine learning has increasingly been applied to biological image analysis, achieving performance comparable to or exceeding that of manual assessments (Doi 2015; Nichols et al. 2019; Wang et al. 2022; Haug and Drazen 2023). In these applications, image segmentation constitutes a critical first step, essential for precise delineation of target structures and accurate determination of morphometric parameters.

Convolutional Neural Networks (CNNs) have emerged as the standard approach for image segmentation, exploiting hierarchical feature extraction and translational equivariance to reliably identify structures regardless of their spatial position (Goodfellow et al. 2016). Among CNN architectures, U-Net is particularly well suited for biomedical images due to its symmetric encoder–decoder design, which integrates high- and low-level features through skip connections to enhance segmentation accuracy. The contracting path extracts and compresses essential spatial information, while the expansive path reconstructs a probabilistic segmentation map in which each pixel is assigned a likelihood of belonging to a specific region (Ronneberger et al. 2015; Oudouar et al. 2025).

Enabling automated morphometric analysis by developing a CNN-based approach for spheroids and organoids segmentation represents a promising strategy to enhance the robustness and efficiency of data analysis in preclinical studies using 3D tumor models. Despite recent advances in automated image tools, most currently available approaches require specialized computational expertise, depend on proprietary software licenses, or rely on workflows involving multiple programming environments and external dependencies. In addition, several available approaches focus exclusively on segmentation, or area-based metrics, without volumetric estimation. True three-dimensional reconstruction typically requires z-stacks and confocal microscopy, which is impractical for high-throughput analysis of large spheroid cohorts. As a result, most available tools, including the present approach, estimate volume from a single two-dimensional image using simplified geometric models. Nonetheless, accurately delineating the spheroid boundary, regardless of its underlying shape, remains an essential prerequisite for reliable diameter extraction and, consequently, for volume estimation.

Spheroid morphology is strongly dependent on the cellular composition and extracellular matrix or scaffolds, with many tumor spheroids exhibiting irregular or elongated shapes (Guillaume et al. 2019). Indeed, recent studies have shown that both spheroids and organoids exhibit a broad spectrum of morphologies, ranging from compact and spherical to loose, irregular, and branched architectures, including distinct phenotypes such as grape-like or stellate structures associated with differences in cell–cell adhesion and invasive behavior (Karolak et al. 2019; Blondeel et al. 2025). Moreover, treatment-induced alterations can further induce 3D structure modifications, leading to degradation, cell dissociation, or migration, which highlights the need for automated and reliable tools to accurately evaluate spheroid parameters under diverse experimental conditions and morphologies.

Here we aimed to develop an automated convolutional neural network (CNN)-based tool for the segmentation and volumetric analysis of tumor spheroids and organoids that exhibit diverse structural and biological characteristics. The model was trained and validated using a total of 615 microscopy images from single-cell-line and hybrid spheroids, as well as organotypic tumor spheroids derived from human melanoma lesions, together comprising a range of different morphological structures. The training dataset included treated and untreated spheroids and organoids exposed to nine therapeutic conditions, consisting of monotherapy and combined treatments, including radiotherapy, small interfering RNA (siRNA), virus-based gene therapy, engineered extracellular vesicles, and their combinations. The U-Net–based CNN achieved high performance, with a Dice Score of 0.9478 and validation accuracy of 98.56%, demonstrating reliable segmentation even across heterogeneous morphologies. Volume values estimated by the model showed a positive correlation with manual measurements from two independent experts, confirming the potential of this approach to enhance precision and efficiency for morphometric assessments in preclinical studies using 3D culture.

## Methods

### Implementation

#### Image acquisition and datasets

Spheroids were cultured in 96-well plates coated with 1% agarose and imaged under bright-field microscopy using EVOS FL (Thermo Fisher Scientific) or DMi8 (Leica Microsystems) microscopes, at 4× magnification. Images were saved at 1280×960 resolution. The dataset included tumor spheroids derived from human (A375, SKMel-28, SKMel-147, UACC-62, WM-1366) and canine (CMGD-5 and TLM-1) melanoma cells, canine osteosarcoma (D-17), human non-small cell lung cancer (A549), triple negative breast cancer (MDA-MB-231), and pancreatic adenocarcinoma (PANC-1) cell lines, as well as human embryonic kidney cells (HEK293-FT), commonly used as a non-tumoral cell control. Hybrid spheroids obtained by co-culturing tumor cells with human THP-1 monocytes or Peripheral Blood Mononuclear Cells (PBMCs) were also included, along with Patient-Derived Organotypic Tumor Spheroids (PDOTS) generated from human melanoma lesions. PDOTS establishment and their use for drug response analysis were conducted under institutional approval by the Local Ethics Committee (CEP no. 031/12) and National Ethics Committee (CONEP no.023/2014).

A total of 615 images were used for model development, divided into 315 images for training, 200 images for supervised validation, and 100 images for unsupervised validation. Among these, 515 images (315 training + 200 supervised validation) were manually annotated to generate ground-truth masks, whereas the 100 images used for unsupervised validation were not annotated. All ground-truth masks were manually generated by a single annotator using the Freehand tool in ImageJ. Dataset division preserved variation in spheroid morphology, size, and density across all subsets. Table 1 shows spheroid diversity and representative images for the training set. Each spheroid was assigned to descriptive categories capturing morphological and experimental heterogeneity. The following attributes were annotated: shape (round or irregular), clustering (loose or compact), budding (present or absent), margin definition (defined or undefined), size (small, medium, or large), and treatment status (treated or untreated). For treated spheroids, a numerical sub-label from 1 to 9 was used to specify the treatment condition applied. These categories included monotherapies and combination approaches involving radiotherapy, engineered extracellular vesicles, small interfering RNAs (siRNAs), and virus-based gene therapy. Numerical labels were used to standardize treatment information across the dataset while avoiding condition-specific bias during model training.

**Table 1:**
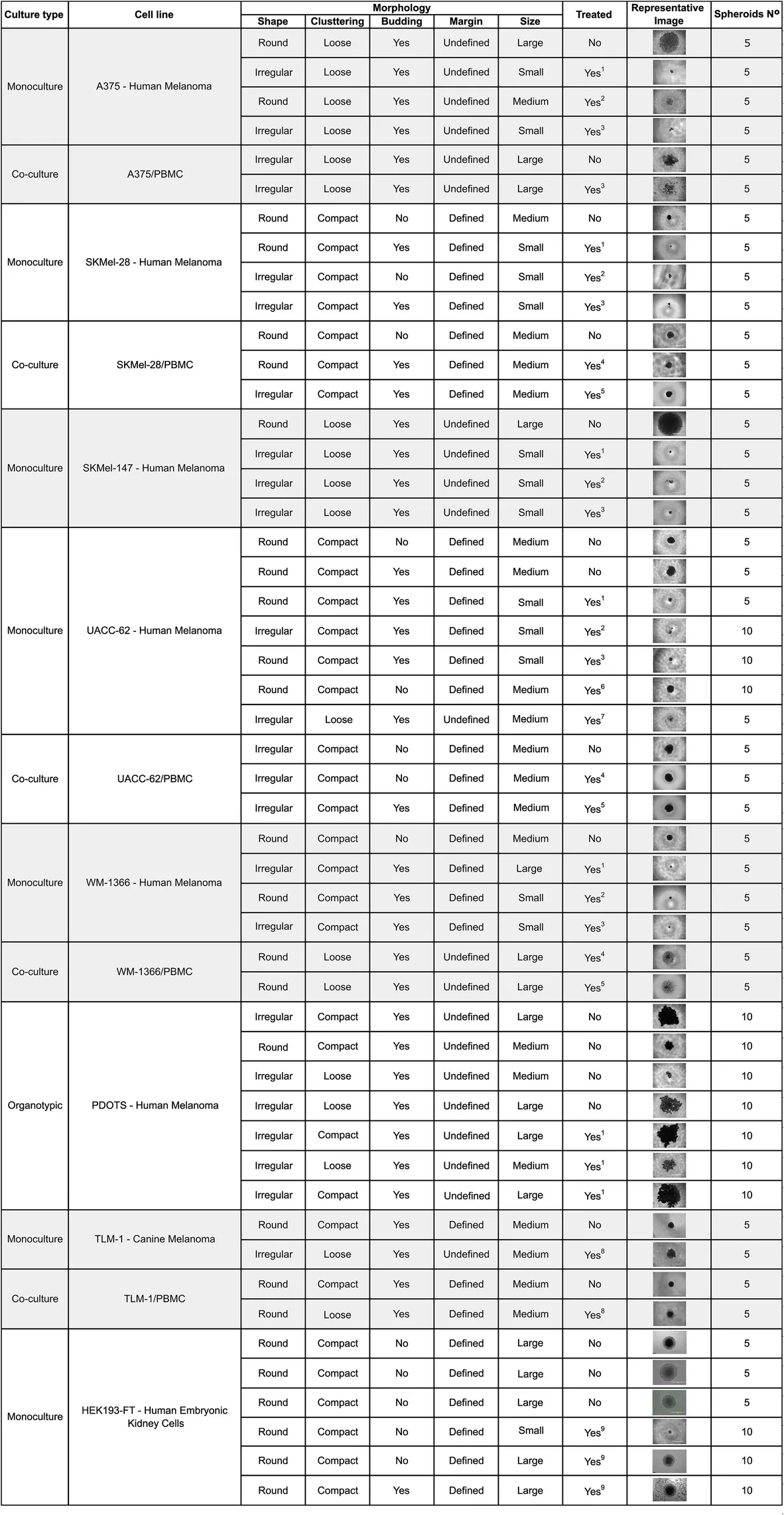
Overview of spheroid diversity included in the training dataset

#### Image preprocessing

For all images requiring ground-truth masks, spheroid contours were first manually annotated in ImageJ using the Freehand tool and binarized to the 0–1 range. For training, images were converted to grayscale, resized to 256×256 pixels, and loaded using EBImage or magick packages to create a uniform input. To enhance contrast and improve the visibility of spheroid boundaries, histogram equalization was applied using the equalize function from the EBImage package (v4.48.0) prior to model input.

#### Neural network architecture and training

**Fig. 1:**
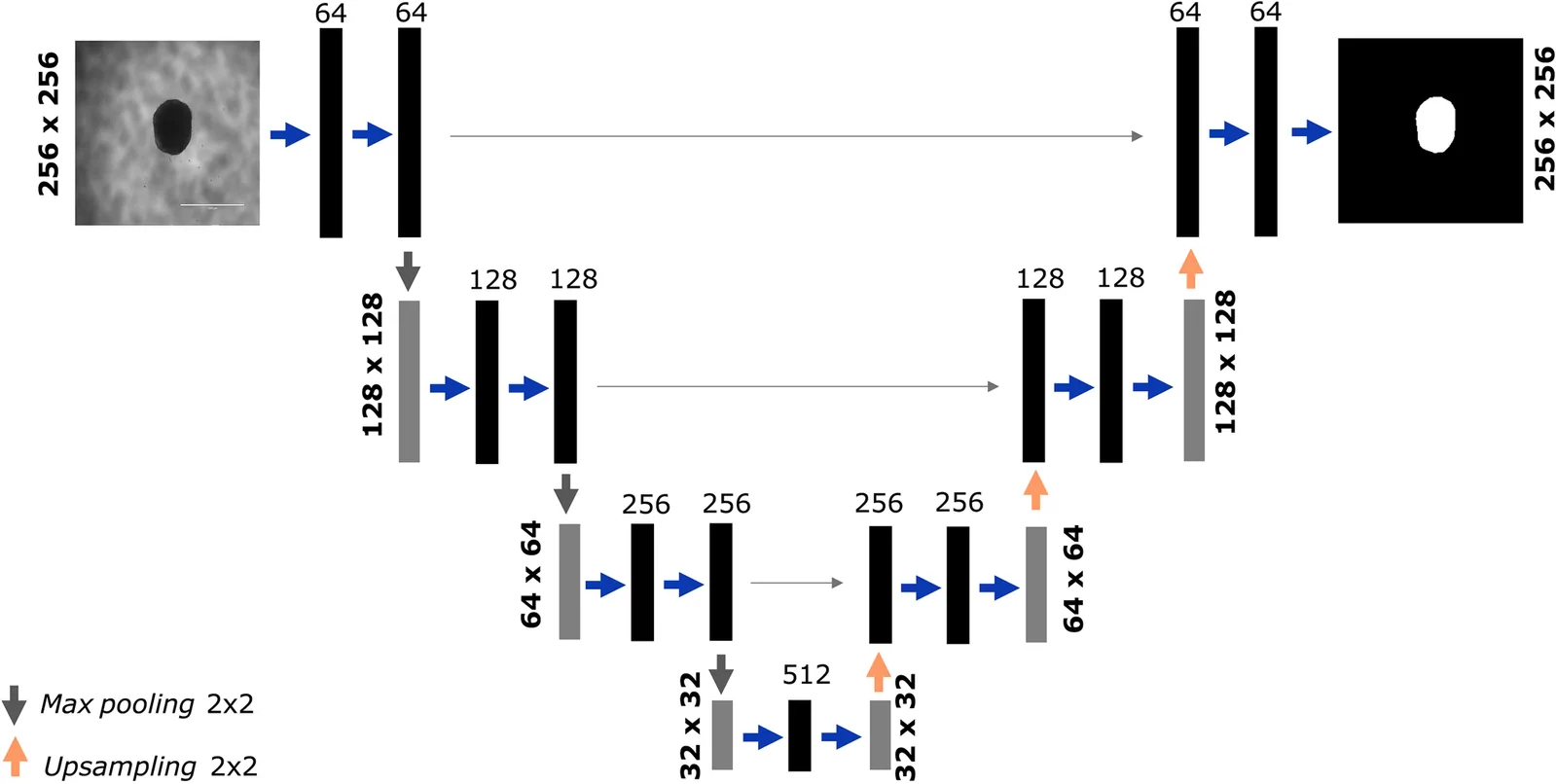
U-Net architecture. U-Net convolutional neural network architecture used for spheroid segmentation, showing the encoder–decoder structure with convolutional blocks, max-pooling, upsampling layers, and skip connections. Scale bar: 1000 µm

The U-Net CNN was implemented in R (v4.4.2) using Keras (v2.15.0) with TensorFlow (v2.16.0) as the backend. The architecture followed the encoder-decoder design described by Ronneberger et al. (2015). As shown in Fig. 1, the encoder contained three convolutional blocks, each composed of two 3×3 convolutions with ReLU activation and same padding, followed by max-pooling for spatial downsampling. The bottleneck consisted of two 3×3 convolutions with 512 filters. The decoder mirrored the encoder through upsampling layers and two convolutional layers per block, with skip connections linking each decoder stage to its corresponding encoder stage. A final 1×1 convolution with sigmoid activation generated a pixel-wise probability map representing the spheroid mask.

The model was trained for 30 epochs using a batch size of 16 and the Adam optimizer with an initial learning rate of 0.0005. Binary cross-entropy was used as the loss function. A learning-rate reduction strategy decreased the learning rate by half when the validation loss did not improve for five consecutive epochs, with a minimum value of 1×10⁻⁶. Fifteen percent of the training data were randomly assigned for internal validation during training using the *set.seed* function to allow reproducibility.

**Fig. 2:**
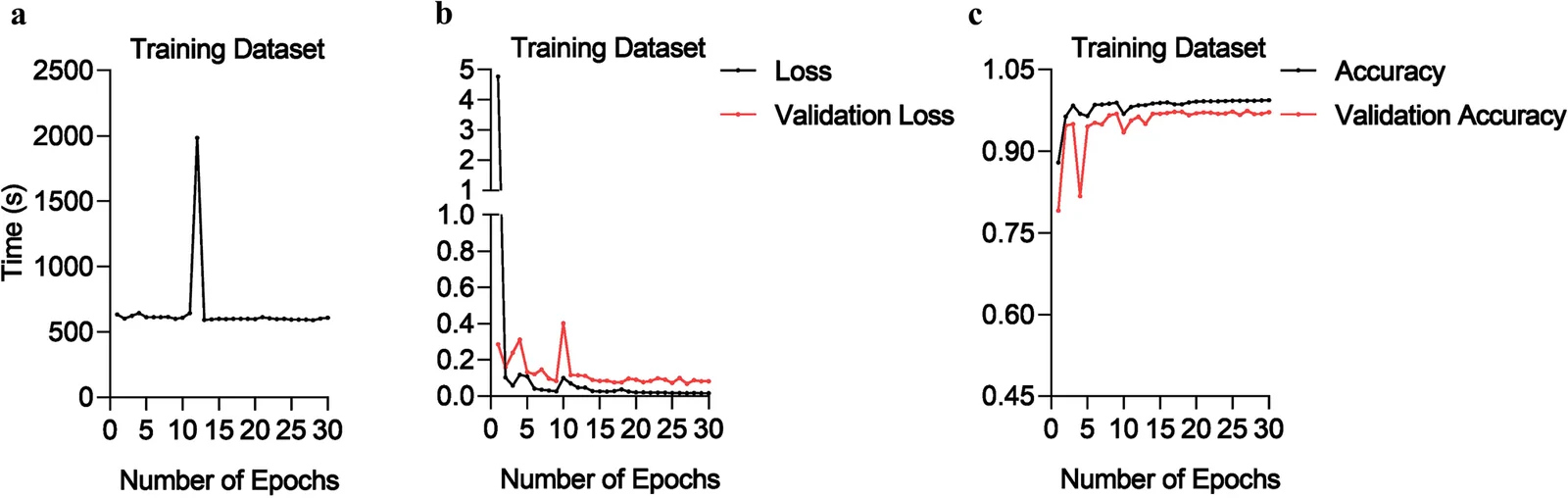
Performance during training. **a**. Training time per epoch. **b**. Training and validation loss curves, and **c**. Training and validation accuracy over 30 epochs. Validation data refers to randomly assigned images for internal validation during training

Training required an average of 600 s per epoch, with the exception of a transient increase to 1,984 s at epoch 12 (Fig. 2a). The training time returned to approximately 600 s in the subsequent epochs, indicating that this increase was isolated and did not persist during training. The initial training loss (4.7683) decreased rapidly during the first epoch, reaching 0.0151 by the final epoch, while validation loss converged to 0.0813 (Fig. 2b). Training accuracy increased from 0.8792 to 0.9935, reaching an internal validation accuracy of 0.9715 on the 15% validation subset by the last epoch (Fig. 2c). These values are in line with or superior to those reported in recent studies applying U-Net to biological image segmentation, including tumor spheroids and skin lesions (Hacıosmanoğlu et al. 2025; Xu et al. 2024). Additionally, the close behavior of training and validation curves indicates no evident overfitting (Ruby et al. 2020).

#### Supervised segmentation validation

**Fig. 3:**
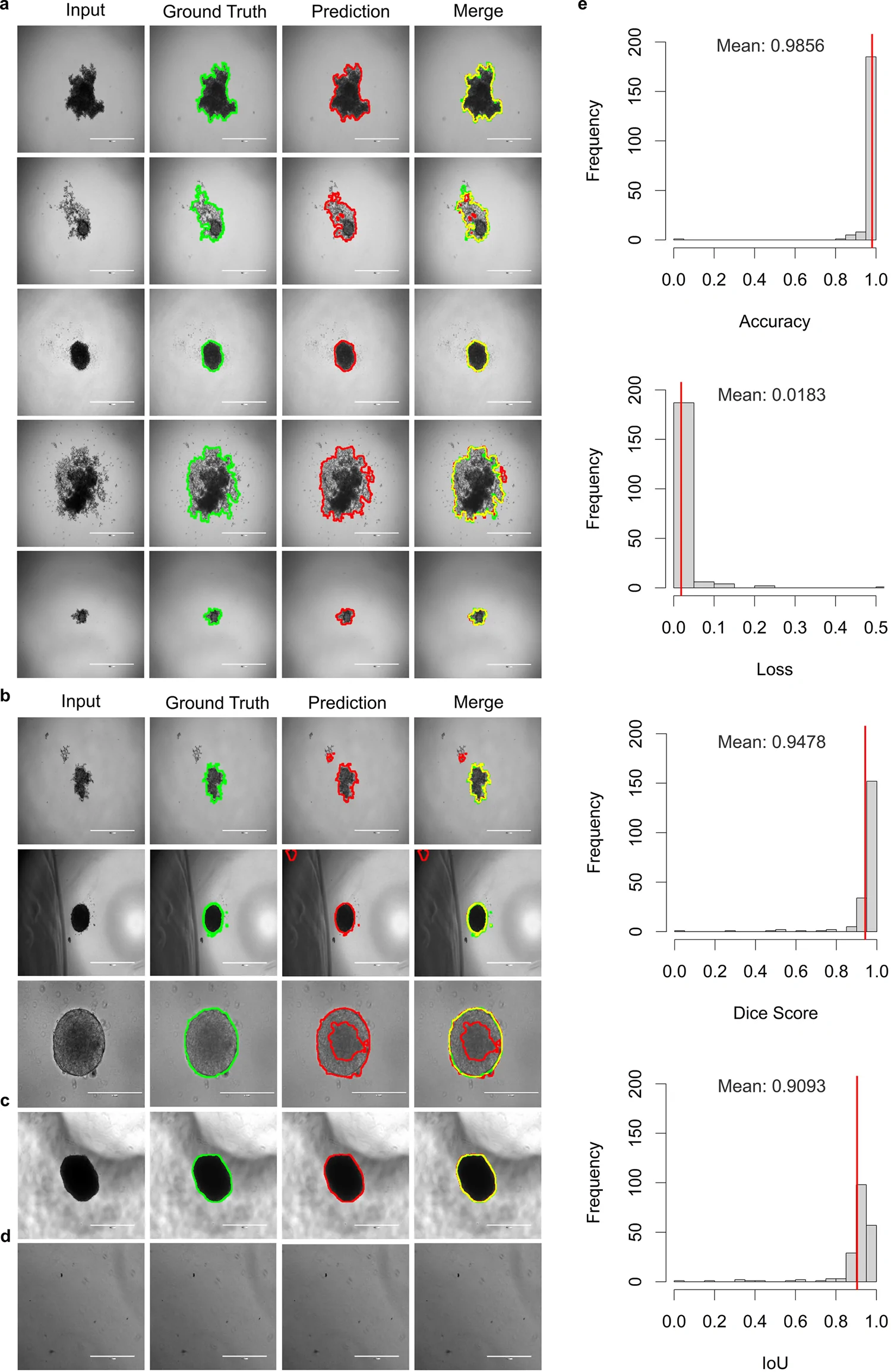
U-Net performance during supervised validation. **a**. Representative examples comparing the input image, ground-truth mask (green), and predicted mask (red). Regions of overlap between ground-truth and predicted segmentations are highlighted in yellow. **b**. Examples of challenging cases leading to segmentation inaccuracies, including misclassification of pixels associated with dissociated cell clusters or irregular background features. **c-d**. Challenging scenarios in which correct segmentation was achieved, including images with heterogeneous background (c) and negative control images lacking spheroids (d). **e**. Quantitative validation metrics, including segmentation accuracy, validation loss, Dice score, and Intersection over Union (IoU). The red vertical line represents the mean value. n=200. Scale bar: 1000 µm

Supervised validation was performed using an independent set of 200 images with corresponding ground-truth masks, composed of 199 spheroids from 13 different cell models and 1 negative control (microscopy with no spheroid). The distribution of spheroids included in the supervised validation dataset across the different cell lines and models is provided in Table S1. Importantly, the validation datasets were organized to include spheroids with distinct morphological characteristics based on independently available spheroid images, rather than to directly compare segmentation performance between individual cell lines. Model predictions were generated as probability maps and subsequently binarized using a threshold of 0.5. For qualitative evaluation, input images were displayed alongside contour-overlaid counterparts: ground-truth contours are shown in green, predicted contours in red, and regions of overlap between the two masks are highlighted in yellow, enabling direct visual comparison of segmentation performance. Representative examples of successful spheroid segmentation are presented in Fig. 3a. More challenging cases included images containing dissociated cell clusters, irregular background artifacts, or spheroids exhibiting heterogeneous density between the core and periphery, characterized by a dark central core, a lighter intermediate region, and a dark peripheral margin (Fig. 3b). To quantitatively assess the segmentation performance associated with this specific morphology, the seven spheroids presenting these characteristics were compared with the remaining 192 spheroids. Notably, all spheroids presenting this morphology were derived from HEK293-FT cells and were associated with reduced segmentation performance overall (Table S2).

Specifically, they showed lower mean Accuracy, Dice, and Intersection over Union (IoU) values (0.9317, 0.6345, and 0.4908, respectively) compared with the remaining spheroids (0.9875, 0.9589, and 0.9241, respectively), as well as higher loss (0.1975 vs 0.0118). Importantly, complex background features did not universally impair model performance, as shown by a representative spheroid correctly segmented despite a challenging background (Fig. 3c). Finally, the negative control image lacking spheroids was correctly classified (Fig. 3d).

Despite challenges, the model showed strong quantitative performance, achieving a mean accuracy of 0.9856 and a validation loss of 0.01813 in this independent dataset of 200 images. Segmentation quality metrics further supported robust agreement between predicted and ground-truth masks, with Dice and IoU scores of 0.9478 and 0.9093, respectively (Fig. 3e), which are consistent to those reported in previous applications of U-Net to biomedical imaging (Ronneberger et al. 2015; Oudouar et al. 2025). Of note, to provide a clear distinction between the datasets used for model evaluation, Table S3 summarizes the performance metrics obtained during training and on the independent supervised validation dataset.

#### Unsupervised validation and generalization

To evaluate model generalization to new data, the trained CNN was applied to an independent set of 100 images without ground-truth masks. Predicted segmentations were evaluated qualitatively through visual comparison with the corresponding input images using the red contour to delineate model predictions. Overall, the model produced coherent segmentations across spheroids derived from various cell lines, co-culture conditions, and treatment groups, including challenging cases with irregular shapes and small sizes (Figure S1). A single false-positive segmentation was observed in a darker background region (highlighted with a light-blue box) in Figure S1, indicating that background features may occasionally impair segmentation. Nevertheless, this unsupervised validation indicated that the model is robust to experimental variability and can be used in independent datasets without retraining.

#### Volume estimation from predicted masks

Volume estimation was performed directly from the binary masks generated by the U-Net model. Following segmentation, in case more than one object was identified, only the largest connected component was considered as the spheroid for subsequent volume estimation. The coordinates of the pixels belonging to this component were then used to derive two morphometric parameters, D_max_ and D_min_, using the Feret-diameter method. D_max_ was defined as the maximum Euclidean distance between two pixels within the segmented spheroid:

$${D}_{max, pixel}={max}_{i,j} \sqrt{{\left({x}_{i}-{x}_{j}\right)}^{2}+{\left({y}_{i}-{y}_{j}\right)}^{2}}$$

where *x*_i_ and *y*_i_ are the horizontal and vertical pixel coordinates of a pixel *i* within the segmented spheroid, and *x*_j_ and *y*_j_ are the coordinates of another pixel *j* within the same region. The notation max_i,j_ indicates that this Euclidean distance is computed for every possible pair of pixels (*i*, *j*) belonging to the segmented region, and the largest such distance is taken as D_max_._pixel_. The resulting pixel distance was converted to micrometers using the image calibration factor:

$$D_{max} = D_{max,pixel}\quad x \left(\frac{1000}{p}\right)$$

where *p* is the number of pixels corresponding to 1000 µm in the image.

In the first implementation, the minor diameter (D_min_) was then estimated using an orthogonal-projection approach to reduce sensitivity to irregular or budding spheroid morphology. The two pixels defining D_max_ established the principal axis of the segmented spheroid, and the perpendicular distance of every segmented pixel to this axis was calculated as:

$$d_i = |(x_i - x_1)\hat{v}_y - (y_i - y_1)\hat{v}_x|$$

where *x*_i_ and *y*_i_ are the coordinates of a given pixel *i* within the segmented spheroid; *x*_1_ and *y*_1_ are the coordinates of one of the two pixels that define the D_max_ axis; and v̂_x_ and v̂_y_ are, respectively, the horizontal and vertical components of the unit vector v̂ pointing along the D_max_ axis. This expression is evaluated for every segmented pixel *i* and returns its perpendicular (orthogonal) distance to the D_max_ axis; D_min_ was defined as the median of these perpendicular distances, converted to micrometers using the same calibration factor:

$${D}_{min}={median(d}_{i}) x (\frac{1000}{p})$$

To avoid biologically implausible underestimation, especially for spheroids with irregular or budding contours, we first sought to add a lower-bound constraint, such that D_min_ could not fall below one-third of D_max_:

$${D}_{min}={max(D}_{min}, (\frac{{D}_{max}}{3})$$

However, because perpendicular distances were calculated across all pixels within the segmented region, the median was strongly influenced by the area distribution of the spheroid. Consequently, the unconstrained D_min_ frequently fell below one-third of D_max_, including for relatively regular spheroids, causing the D_max_/3 constraint to determine D_min_ in most cases.

To address this limitation, the D_min_ calculation was then updated to derive the minor diameter from the segmented area using an equivalent-ellipse assumption. Specifically, the segmented area was calculated from all pixels belonging to the selected spheroid and converted to physical units using the original-image calibration. Assuming the major axis corresponds to D_max_, D_min_ was calculated from the ellipse area relationship:

$$Area\left(A\right)=\frac{\pi }{4}{D}_{max}x{D}_{min}$$

and therefore:

$${D}_{\mathrm{m}\mathrm{i}\mathrm{n}}=\frac{4A}{\pi {D}_{\mathrm{m}\mathrm{a}\mathrm{x}}}$$

Lastly, a geometric upper bound was implemented such that D_min_ could not exceed D_max_.

Importantly, because the original bright-field images (1280×960 pixels) were resized to a fixed 256×256 input, distorting the aspect ratio of the segmented spheroid within the mask, we noticed that the metrics obtained depended on the orientation of the spheroid within the image, rather than solely on its true physical dimensions (an identical spheroid imaged at a different rotation could yield a different estimated volume). This problem was solved by mapping mask pixel coordinates back to the original image's pixel grid before computing any metrics, using separate scaling factors for each axis:

$${s}_{X}= \frac{W}{256} , {s}_{Y}= \frac{H}{256}$$

where *W* and *H* are the width and height, in pixels, of the original (pre-resize) image. Each pixel coordinate (*x*_mask_, *y*_mask_) in the segmented mask was accordingly converted to its corresponding position in the original image as:

$$x={x}_{mask} .{ s}_{x} , y={y}_{mask} .{ s}_{y}$$

**Fig. 4:**
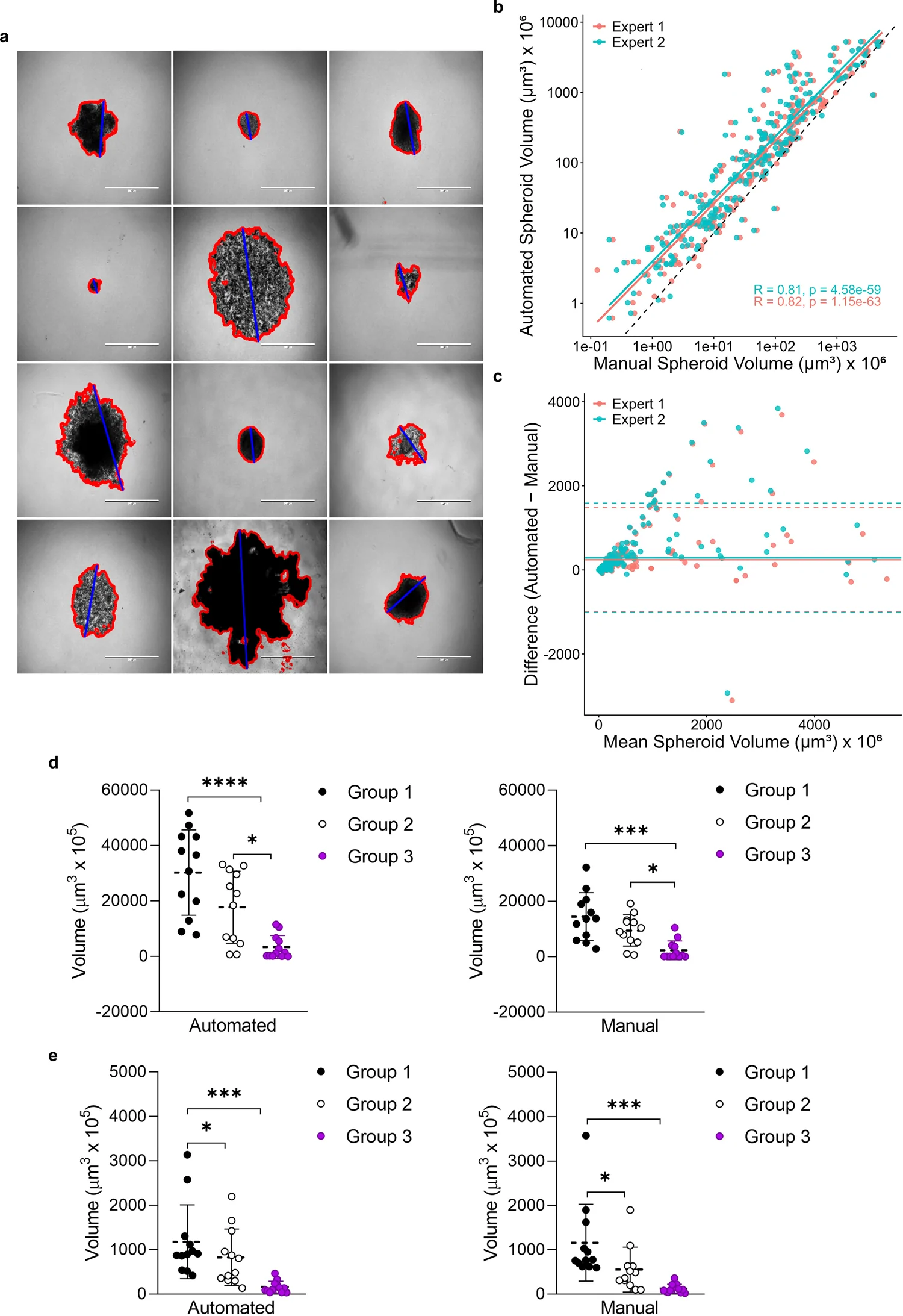
Unsupervised validation and comparison between automated and manual spheroid volume measurements. **a**. Representative bright-field images overlaid with the predicted spheroid contour (red) and the maximum Feret diameter (Dmax, blue). **b**. Scatter correlation plot with linear regression and Pearson correlation analysis. **c**. Bland–Altman plot showing the differences between SpheroQuaNNt and manual volume measurements plotted against their mean. The solid line represents the mean difference (bias), and the dashed lines represent the 95% limits of agreement (LoA). **d-e**. Comparison of automated and manual spheroid volume estimates across two independent experimental datasets containing untreated and treated spheroids under different experimental conditions. Each group included n = 12 spheroids. **d**: A375 and **e**: SKMel-28 cell line. Scale bar: 1000 µm. Data are presented as mean ± SD. Statistical differences were analyzed by One-way ANOVA and Tukey’s post hoc test. * p<0.05 ***p<0.001 ****p<0.0001

For visual inspection of model performance, along with the red contour outlines overlaid on the input images, we added a blue axis line corresponding to the measured maximum diameter (D_max_), allowing rapid verification of the geometry used for volume estimation (Fig. 4a), which was calculated using a semi-ellipsoidal approximation commonly applied in both in vitro and in vivo tumor models (Tomayko-Reynolds, 1989), where:

$$Volume \quad (V) = 0.5 \times D_{\min}^2 \times D_{\max} \ [\mu\mathrm{m}^3].$$

This formula assumes the spheroid approximates a prolate ellipsoid (ellipsoid of revolution), defined by one major axis (D_max_) and two equal minor axes (D_min_). This is equivalent to assuming rotational symmetry around the D_max_ axis: the spheroid's cross-sectional width perpendicular to D_max_ is assumed constant and equal to D_min_. Thus, this approach provides an estimated volume from a single 2D bright-field image and therefore relies on the assumption that the measured 2D dimensions adequately represent the three-dimensional spheroid geometry.

Quantitative per-image results, including D_min_, D_max_, and V values, were used for downstream statistical analysis and comparison with manual measurements.

#### Comparison with manual measurements

To evaluate the performance of the automated approach, a set of 270 predicted masks was used to calculate spheroid volumes and compare them with measurements obtained from two independent experts. As detailed in 2.1.6, during the course of this study, we first tested a volume-estimation approach in which D_min_ was calculated from the median of orthogonal distances to the Dmax axis, subject to a lower-bound constraint of D_max_/3. This first implementation (SpheroQuaNNt v.1) was used to estimate volumes for comparison with manual measurements, presented in Supplementary Figure S2. The results showed a positive correlation between automated and manual measurements for both experts (Fig. S2b), together with agreement between methods on Bland–Altman analysis (Fig. S2c) and preservation of statistical differences between groups in two additional independent datasets (Fig. S2d–e). However, we noticed that the D_max_/3 constraint was determining D_min_, regardless of spheroid morphology, rather than functioning only as a safeguard against extreme irregularity.

To overcome this limitation, we updated the calculation to derive the minor diameter from the segmented area under an equivalent-ellipse assumption. Using this corrected implementation (SpheroQuaNNt v.2), automated and manual volumes remained positively correlated for both experts (Pearson r=0.82, p=1.15×10^–63^ and r=0.81, p= 4.58 × 10^–59^, for experts 1 and 2, respectively; Fig. 4b). Bland–Altman analysis showed a mean bias (automated − manual) of 244.61 µm3 ×10^6^ for Expert 1 and 289.42 µm3 x10^6^ for Expert 2, with 95% limits of agreement of −990.6 to 1,479.83 µm3 ×10^6^ (Expert 1) and −1,008.71 to 1,587.56 µm3 x10^6^ (Expert 2) (Fig. 4c). As shown in Fig. 4c, the difference between automated and manual measurements was small and consistent for smaller spheroids and became progressively larger as spheroid volume increased.

To assess external robustness, two additional independent datasets were analyzed, corresponding to two separate experiments containing three spheroid groups. These datasets were used to compare statistical differences between groups obtained from automated and manual measurements. Although automated measurements produced overall larger absolute volume estimates than manual annotation, the automated analysis preserved the relative differences between spheroid groups (Fig. 4d–e), indicating that the method reliably captures biologically relevant variations in spheroid size. Together, these results suggest that, even when absolute values differ, the model can be reliably used to detect biologically relevant differences between experimental conditions.

#### Statistical analysis

Statistical analyses were performed in R (v. 4.5.1). Pearson’s correlation was assessed using the cor.test function in base R. Agreement between automated and manual measurements was assessed using Bland–Altman analysis, with the mean bias and 95% limits of agreement calculated as the mean difference ± 1.96 standard deviations of the differences. Comparisons among the three groups in the external validation datasets were performed using GraphPad Prism, with one-way analysis of variance (ANOVA) followed by Tukey’s multiple-comparison post hoc test. A p-value < 0.05 was considered statistically significant.

### SpheroQuaNNt

A user-friendly Shiny application was developed to allow users to perform spheroid segmentation and volume estimation using the trained CNN. The app is publicly available at https://nlealdovaizem.shinyapps.io/SpheroQuaNNt-v2/ and provides a step-by-step interface where users can upload microscopy images, visualize predicted masks, and estimated volumes.

The application is currently deployed on shinyapps.io, which offers up to 1 GB of RAM for the free-tier environment. Consequently, large batch uploads or memory-intensive image processing may exceed the available resources and lead to temporary interruptions. To overcome these limitations when processing large datasets, users may download the source code and run the application locally, where performance depends on the available hardware resources. Future developments will focus on reducing memory consumption and improving computational efficiency.

#### User manual

The application provides an intuitive interface for spheroid segmentation and automated volume estimation. Users may upload one or multiple bright-field microscopy images by clicking the **Browse** button under **Upload Microscopy Images**. Images should ideally display a single spheroid within the field of view; however, if more than one spheroid is present, the application automatically identifies all objects and computes the volume only for the largest spheroid.

Image calibration is performed using the scale bar present in the original image. The application automatically detects the scale bar, after which the user specifies its physical length (e.g., 1000, 500, or 250 µm). The corresponding pixel-to-micrometer calibration is then calculated automatically. If the scale bar cannot be detected, the calibration can be entered manually as a fallback. Although images are automatically resized to 256 × 256 pixels for processing by the U-Net model, calibration is based on the original image and is used to convert the mask-derived dimensions from pixels to micrometers and calculate the estimated volume in µm3. Upon clicking **Run Segmentation**, each image is automatically preprocessed and passed through the U-Net model. The predicted spheroid contour is displayed as a red outline merged on the original grayscale image, allowing immediate visual inspection of segmentation accuracy. To support verification of geometric measurements, the longest Feret diameter is shown as a blue line, corresponding to the major axis used in volume estimation. Results can be downloaded by clicking **Download Results (ZIP)** or **Download Table (CSV)**.

##### Supported image formats and requirements

The application accepts the following image formats: TIFF, JPEG, and JPG. Images should preferably contain a single spheroid per frame. The recommended resolution is 256×256 pixels, corresponding to the resolution used during model training. If a different resolution is used, images will automatically be resized prior to model loading. Images should present adequate contrast between the spheroid and the background.

#### Data handling

As a Shiny application, SpheroQuaNNt does not store, archive, or retain user-uploaded images or other user-generated data. Uploaded images are used only during the active Shiny session for segmentation and volume estimation and are not incorporated into any database or training dataset. No user-identifiable information is requested by the application. Thus, the application is designed to process the uploaded images without persistent storage of user data.

### Comparison with existing tools

To place the proposed workflow in the context of currently available approaches for spheroid morphometric analysis, previously reported tools were compared according to segmentation strategy, use of learning-based methods, volume estimation approach, and methodological assumptions, as well as interface accessibility (Table 2).

**Table 2:** Comparison of existing tools for spheroid morphometric analysis

Tool	Interface	Segmentation approach	Learning-based	Volume estimation	Core assumption	Reference
AnaSP	Web-based	Histogram-based intensity thresholding (coarse-to-fine)	No	Yes (voxel-based 2D-to-3D)	Smooth boundaries, strong edges, spherical geometry	Piccinini et al. (2015)
SpheroidSizer	Desktop	Active contour (snake) segmentation guided by image gradients	No	Yes (estimation from 2D major/minor axes)	Histogram separability, local spherical symmetry	Chen et al. (2014)
ReViMS	Desktop	Intensity-based surface extraction from fluorescence z-stacks	No	Yes (z-stack based)	z-stack-based 3D reconstruction	Piccinini et al. (2017)
OrganoSeg	Desktop	Morphological open–close filtering with multi-scale adaptive thresholding (Otsu-based)	No	No (area-based metrics)	N/A	Borten et al. (2018)
SpheroScan	Web-based	Convolutional Neural Network (Mask R)	Yes	No (area-based metrics)	N/A	Akshay et al. (2023)
SpAi	Desktop	Convolutional Neural Network (U-Net)	Yes	No (radius-based metrics)	N/A	Hacıosmanoglu et al. (2025)
SpheroQuaNNt	Web-based/Desktop	Convolutional Neural Network (U-Net)	Yes	Yes (estimated from mask-derived Feret diameters)	Data-driven feature learning for segmentation, semi-ellipsoidal geometric model used for volume estimation	This study

As shown in Table 2, most existing tools rely on predefined intensity thresholds, edge contrast, or user-guided segmentation to delineate spheroid boundaries (Chen et al. 2014; Piccinini et al. 2015, 2017; Borten et al. 2018), which can limit robustness when applied to spheroids with heterogeneous density, irregular morphologies, or low-contrast images.

More recent learning-based approaches employing convolutional neural networks have improved segmentation robustness across variable imaging conditions while reducing dependence on manual processing. However, these methods remain primarily focused on two-dimensional morphometric outputs, including area (Akshay et al. 2023) and radius (Hacıosmanoglu et al. (Hacıosmanoğlu, et al. 2025)) measurements. In contrast, SpheroQuaNNt combines automated U-Net–based segmentation with volumetric estimation derived from mask-based morphometric parameters. By directly learning contour features from microscopy images, the proposed model enables accurate boundary detection across a broad spectrum of spheroid and organoid morphologies without relying on predefined intensity distributions or explicit geometric shape assumptions. In addition, the platform provides an accessible web-based and desktop-compatible interface for automated morphometric analysis, facilitating its application across diverse experimental settings, while avoiding dependence on proprietary computational environments that may limit accessibility in research settings without dedicated software licenses.

To assess segmentation performance relative to an existing open-source approach applicable to bright-field images, we compared SpheroQuaNNt with the ImageJ macro developed by Guyon et al. (2020). Because this macro performs two-dimensional area-based segmentation and does not provide volumetric measurements, we adapted our app to output spheroids’ area. The same set of 30 images, representing five cell lines (A375, A549, MDA-MB-231, SKMel-28, and UACC-62) under different culture conditions, was analyzed using both approaches. As shown in Figure S3a, the ImageJ macro successfully segmented 6/30 spheroids (20%), primarily corresponding to relatively loose spheroids without pronounced density heterogeneity. For 7/30 spheroids (23%), particularly those exhibiting heterogeneous density, the macro fragmented a single spheroid into multiple detected objects. A further 13/30 spheroids (43%) were not detected, while 4/30 (13%) were only partially segmented, mostly capturing fragments of the spheroid boundary. In contrast, SpheroQuaNNt successfully segmented all 30 spheroids in this comparison subset.

Although the ImageJ macro allows multiple images to be processed in a single batch for area measurement, in our experience, the scale calibration was not consistently retained across images, requiring individual calibration for each sample. Importantly, for spheroids that were correctly segmented, the resulting 2D area measurements were comparable for both methods, indicating that the primary difference between the approaches was segmentation reliability rather than systematic differences in area estimation (Figure S3b).

## Discussion

Three-dimensional cell culture systems have become increasingly important in preclinical research because they better recapitulate tumor architecture, microenvironmental gradients, and therapy response compared to traditional 2D monolayers (Barbosa et al. 2021). Tumor spheroids and patient-derived organotypic models provide more physiologically relevant platforms for evaluating treatment efficacy, drug penetration, radiation response, and cellular heterogeneity (Hong et al. 2021). However, despite their scientific value, quantitative analysis of spheroid volume remains a major bottleneck as manual measurements are labor-intensive, subjective, and difficult to scale for high-throughput studies. Automated and reliable computational tools therefore play a crucial role in enabling standardized and reproducible analysis.

In this work, we developed a U-Net-based convolutional neural network capable of segmenting spheroids and organoids from bright-field images with high accuracy and estimating their volumes. Importantly, the present study used a heterogeneous dataset, including spheroids derived from eleven human and canine tumor cell lines, human embryonic kidney cells, co-culture systems with immune cells, and patient-derived organotypic tumor spheroids. In addition to the diversity of cell lines and culture conditions, the dataset included nine distinct treatment conditions that reflect the experimental interventions used in different studies, including radiotherapy, engineered extracellular vesicles (EVs) carrying different siRNA cargo; siRNA-based interventions using commercial lipophilic or polymeric macromolecules, and virus-mediated gene therapy. Training and validating the model across these nine treatment groups exposed it to a wide range of therapy-induced morphologies, such as altered compactness, changes in margin definition, budding, and variations in core density, thereby helping the network learn features relevant to both untreated and treatment-modified spheroids and improving its capacity to generalize to experimentally perturbed samples.

During supervised validation, the proposed model achieved a Dice score of 0.9478 and an IoU of 0.9093, with minimal divergence between training and validation losses, indicating stable learning and no evident overfitting. These metrics are comparable to those reported in previous U-Net–based applications for spheroid and biomedical image segmentation. For example, Hacıosmanoğlu et al. (2025) trained a U-Net model using 291 spheroid images and reported a training accuracy of 89.4% with a loss of 0.0828, while Xu et al. (2024) achieved IoU ranging from 67 to 82% when applying U-Net architectures to skin cancer lesion segmentation. In the present study, the final validation loss was 0.0183, accompanied by a validation accuracy of 0.9856, indicating a low prediction error and accurate pixel-wise classification. The high Dice and IoU values further demonstrate strong spatial agreement between predicted and ground-truth masks, consistent with the ranges typically reported for biological image segmentation, where Dice scores between 0.87 and 0.95 and IoU values between 0.77 and 0.92 are commonly observed (Ronneberger et al. 2015; Xu et al. 2024; Oudouar et al. 2025; Sheikh et al. 2025).

Beyond segmentation accuracy, an important distinction between SpheroQuaNNt and previously reported spheroid analysis tools is its accessibility and the diversity of the data used for model development. SpheroQuaNNt is provided as a free, user-friendly application, making automated spheroid segmentation and volume estimation accessible without specialized commercial software or programming expertise. In addition, the model was trained and validated using a heterogeneous dataset composed of spheroids with diverse morphologies, cell origins, culture conditions, and treatment responses. Such diversity is particularly relevant for automated image analysis, as treatment-induced changes in compactness, boundary definition, budding, and density can substantially affect spheroid morphology and consequently, segmentation performance. The ability to accurately segment spheroids across this range of morphological characteristics is therefore an important aspect of the practical utility of the tool. This was further illustrated by the comparison with an established ImageJ macro, in which only 20% of the spheroids in the test dataset (n = 30) were successfully segmented using the macro, whereas SpheroQuaNNt successfully segmented all spheroids.

Accurate delineation of the spheroid boundary is particularly relevant when segmentation is used not only for visualization but also as the basis for quantitative measurements. In SpheroQuaNNt, the segmented mask provides the morphological information required to determine Dmax and Dmin, which are subsequently used for volume estimation. The reliability of these measurements is therefore directly linked to the quality of the segmentation.

Based on these mask-derived measurements, SpheroQuaNNt estimates spheroid volume using a semi-ellipsoidal approximation (Tomayko and Reynolds 1989), since a true three-dimensional volume cannot be directly obtained from a single two-dimensional bright-field image. This type of geometric approximation is analogous to the long-standing use of caliper-based measurements to estimate tumor volume in vivo, where tumors likewise rarely conform to a perfect ellipsoidal shape. The use of such an approximation therefore represents an inherent limitation of inferring volume from diameter-based measurements rather than a limitation specific to SpheroQuaNNt. Accordingly, the two sources of uncertainty should be distinguished: accurate segmentation is necessary to reliably capture the dimensions of the spheroid, whereas the subsequent conversion of these dimensions into volume remains dependent on the underlying geometric assumption. For highly irregular morphologies, such as grape-like or stellate spheroids with pronounced protrusions, volume estimates may therefore be less representative because these shapes do not closely match the assumed geometry. Despite this limitation, comparison with manual measurements performed by two independent experts confirmed the reliability of the automated approach, showing high correlation and preservation of statistical differences between experimental groups, even when absolute values differed.

Finally, we translated our pipeline into a user-friendly Shiny application, SpheroQuaNNt, enabling rapid segmentation and volume quantification without requiring programming expertise or a dedicated software license. The tool allows researchers to analyze individual or multiple images, visualize predicted contours and D_max_ measurements, and export quantitative outputs. By combining a free and accessible interface with a model developed using diverse spheroid morphologies and experimental conditions, SpheroQuaNNt provides a practical approach for standardized quantitative analysis of 3D preclinical models.

Importantly, the diversity of the dataset supports the potential applicability of SpheroQuaNNt beyond the specific models evaluated in this study. Although the organoid models included in this study were derived from melanoma, the broad morphological diversity represented across the training and validation datasets suggests that the model may also be applicable to other organoid models with similar morphological features. Nevertheless, this possibility remains to be experimentally validated.

### Limitations

While SpheroQuaNNt demonstrated robust performance across a heterogeneous dataset, some considerations define the current scope of the model. The CNN showed reduced segmentation performance for spheroids exhibiting pronounced core–periphery density heterogeneity, particularly the dark core–lighter intermediate region–dark peripheral margin phenotype observed in HEK293-FT spheroids, indicating that this morphology remains more challenging for the current model. Volume estimation relies on a semi-ellipsoidal approximation from two-dimensional diameter measurements and may therefore be less representative for highly irregular spheroid morphologies. In addition, the training dataset was acquired using a single magnification and bright-field microscopy modality, and performance under other imaging conditions remains to be established. The morphological diversity represented in the training dataset provides a rationale for expecting SpheroQuaNNt to be applicable to organoids exhibiting diverse morphological features; however, this broader applicability has not yet been experimentally evaluated.

## Conclusions

In this study, we developed and validated a U-Net–based convolutional neural network for automated segmentation and morphometric analysis of tumor spheroids from bright-field microscopy images. The model demonstrated fast convergence during training and strong generalization performance, achieving high validation accuracy and Dice scores, indicating reliable pixel-wise segmentation. While performance was robust overall, spheroids exhibiting marked heterogeneity between core and peripheral regions remained more challenging, highlighting intrinsic limitations associated with complex optical density patterns.

Comparison between automated and manual volume estimations revealed systematic differences in absolute values, with a tendency toward higher volumes predicted by the automated pipeline, especially for larger spheroids. Importantly, however, statistically significant differences between experimental groups were consistently preserved regardless of the measurement approach, supporting the reliability of the proposed method for comparative analyses across experimental conditions.

Overall, this work demonstrates that CNN-based image analysis provides a robust and scalable alternative to manual spheroid quantification, reducing subjectivity and processing time while preserving biologically meaningful differences between experimental conditions. By combining automated segmentation with volume estimation in a free and user-friendly application, SpheroQuaNNt offers a practical tool for standardized quantitative analysis of 3D spheroid and organoid models.

## Supplementary Information

Below is the link to the electronic supplementary material.

Supplementary file 1 

## Data Availability

The application source code, as well as the trained CNN model, are available on GitHub at https://github.com/nleald/SpheroQuaNNt. Datasets and additional information are available from the corresponding author upon request.
