# Supplementary material for: SpheroQuaNNt: U-Net–based volume estimation in tumor spheroid and organoid models for assessing preclinical treatment response
Source: Cytotechnology. 2026 Oct 25;78(6):221. doi: 10.1007/s10616-026-01074-9 (PMC13696662; doi:10.1007/s10616-026-01074-9)
Supplement: Supplementary file 1 [file 10616_2026_1074_MOESM1_ESM.docx]

**Supplementary Information**

**SpheroQuaNNt: U-Net–Based Volume Estimation in Tumor Spheroid and Organoid Models for Assessing Preclinical Treatment Response**

*Nathalia Leal Dovaizem^1,2*^; Jean Carlos dos Santos da Luz^1,2^; Alexis Germán Murillo Carrasco^1,2^; and Luciana Nogueira de Sousa Andrade^1,2^;*

^1^ Center for Translational Research in Oncology (LIM24), Instituto do Cancer do Estado de Sao Paulo (ICESP), Hospital das Clinicas da Faculdade de Medicina da Universidade de Sao Paulo (HCFMUSP), São Paulo 01246-000, Brazil.

^2^ Comprehensive Center for Precision Oncology, Universidade de São Paulo, São Paulo 01246- 000, Brazil.

^*^Corresponding author

**Figure S1.** Qualitative unsupervised validation dataset.

Bright-field images from an independent set of 100 spheroids used for qualitative assessment of model generalization. Each image is displayed with the predicted segmentation contour overlaid in red. This dataset was composed of spheroids from A375 (n = 14), A549 (n = 7), CMGD-2 (n = 5), D17 (n = 4), MDA-MB-231 (n = 4), melanoma PDOTS (n = 9), SKMel-147 (n = 18), SKMel-28 (n = 10), THP-1 (n = 6), UACC-62 (n = 8), and WM-1366 (n = 15). The number of spheroids represented for each model reflects the available independent images and was not selected to enable comparative performance analysis between cell models. Light-blue box highlights a false positive segmentation. Scale bar: 1000 µm.

**Figure S2.** Unsupervised validation and comparison between automated and manual spheroid volume measurements obtained with the initial software implementation.

**a.** Representative bright-field images overlaid with the predicted spheroid contour (red) and the maximum Feret diameter (Dmax, blue). **b**. Scatter correlation plot with linear regression and Pearson correlation analysis. **c**. Bland–Altman plot.  **d-e**. Comparison of automated and manual spheroid volume estimates across two independent experimental datasets comprising untreated and treated spheroids under different experimental conditions. Each group included n = 12 spheroids. Scale bar: 1000 µm. Data is presented as mean ± SD. Statistical differences were analyzed by One-way ANOVA and Tukey’s post test.

**Figure S3.** Comparison of spheroid segmentation by the ImageJ macro and SpheroQuaNNt.

**a.** Bright-field images of 30 spheroids segmented using the ImageJ macro developed by Guyon et al. (2020) and SpheroQuaNNt. For both approaches, the predicted spheroid contour is shown in red and overlaid on the corresponding input image. Colored boxes indicate the segmentation outcome obtained with the ImageJ macro: green, correctly segmented spheroids; red, spheroids not detected/segmented; orange, poorly segmented in which only part of the spheroid was detected; and yellow, spheroids incorrectly segmented into multiple objects. The corresponding SpheroQuaNNt predictions are shown alongside the ImageJ results for direct visual comparison. Scale bar: 1000 µm. **b.** Paired dot plot comparing 2D spheroid area measurements obtained using the ImageJ macro and SpheroQuaNNt. Each pair of points represents the same spheroid analyzed by the two methods (n=30).

**Table S1.** Cell models and number of spheroids included in the supervised validation dataset.

| **Cell line** | **Spheroids (n)** |
| --- | --- |
| **A375** | 10 |
| **A549** | 21 |
| **D-17** | 20 |
| **HEK293-FT** | 7 |
| **MDA-MB-231** | 5 |
| **PANC-1** | 10 |
| **PDOTS** | 13 |
| **SKMel-147** | 37 |
| **SKMel-28** | 29 |
| **THP-1** | 3 |
| **TLM-1** | 17 |
| **UACC-62** | 22 |
| **WM-1366** | 5 |

**Table S2.** Segmentation performance according to spheroid density and margin morphology

| **Dataset** | **Spheroids (n)** | **Accuracy** | **Loss** | **Dice** | **IoU** |
| --- | --- | --- | --- | --- | --- |
| Core–periphery heterogeneity with dark peripheral margin | 7 | 0.9317 ± 0.035 | 0.1975 ± 0.158 | 0.6345 ± 0.203 | 0.4908 ± 0.207 |
| Remaining spheroids | 192 | 0.9875 ± 0.023 | 0.0118 ± 0.019 | 0.9589 ± 0.047 | 0.9241 ± 0.067 |

Values are presented as mean ± standard deviation (SD).

**Table S3**. Summary of SpheroQuaNNt model performance across training and validation datasets.

| **Dataset** | **Spheroids (n)** | **Accuracy** | **Loss** | **Dice** | **IoU** |
| --- | --- | --- | --- | --- | --- |
| Training | 315 | 0.9935 | 0.0151 | - | - |
| Validation during Training |  | 0.9715 | 0.0813 | - | - |
| Supervised Validation | 200 | 0.9856 | 0.01813 | 0.9478 | 0.9093 |
